# Vortioxetine as an Adjunctive Treatment in Schizophrenia: A Systematic Review of Effects on Quality of Life, Anhedonia, Cognitive Function, and Symptom Domains

**DOI:** 10.1192/j.eurpsy.2025.2220

**Published:** 2025-08-26

**Authors:** D. F. Holanda, F. R. de Lima, B. S. Pinto, T. N. Vianez, J. H. P. Raid, E. C. Barbosa

**Affiliations:** 1 Medicine, Federal University of Amazonas, Manaus; 2 Independent practice, São Paulo; 3 Neurology, Federal University of Amazonas, Manaus; 4 Medicine, Evangelical University of Goiás, Anapolis, Brazil

## Abstract

**Introduction:**

Schizophrenia is a severe psychiatric disorder characterized by disturbances in perception, thinking, affect, behavior, and negative symptoms. Depression in patients with schizophrenia worsens disease outcomes by increasing suicide risk, complicating the clinical picture, and reducing social functioning quality. Treatment is challenging, as monotherapy with modern antipsychotics is not always successful. Adding antidepressants may improve outcomes, but the effectiveness of such augmentation often requires further evidence.

**Objectives:**

This study aimed to examine the effects of combining second-generation antipsychotics (SGA) with vortioxetine, a novel multimodal serotonergic antidepressant, on various aspects, including quality of life, anhedonia, cognitive function, and overall symptom improvement in schizophrenia patients.

**Methods:**

We conducted a comprehensive search of PubMed, Embase, Cochrane and Web of Science databases up to September 2024 for studies using Vortioxetine with standard treatments for schizophrenia.

**Results:**

We screened 371 studies and our review included six studies gathering 508 patients. Study type, sample sizes, and follow-up time varied across studies (Figure 1). All studies involved adding Vortioxetine to existing antipsychotic treatments, with dosages ranging from 5-20 mg/day. Study durations varied from eight to 48 weeks. Common scales across multiple studies included: PANSS (Positive and Negative Syndrome Scale), WHOQOL-BREF (World Health Organization Quality of Life Assessment), CDSS (Calgary Depression Scale for Schizophrenia), and various cognitive function tests (e.g., WCST, Verbal Fluency Test, Stroop Task). Overall, the studies reported positive effects of vortioxetine in schizophrenia patients (Figure 2): Improved quality of life, Reduced anhedonia, Enhanced cognitive function, Improved depressive symptoms, Reduced negative symptoms. Most studies reported good tolerability of vortioxetine with minimal side effects.

**Image 1:**

**Figure:**
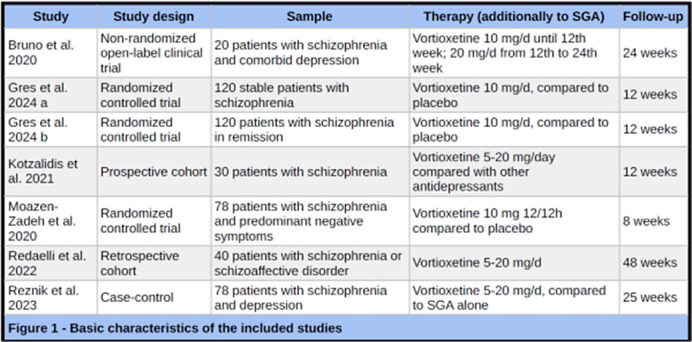

**Image 2:**

**Figure:**
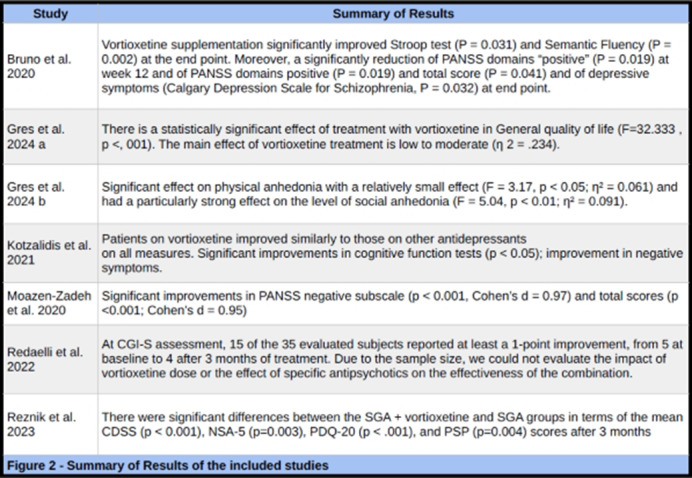

**Conclusions:**

The findings suggest that Vortioxetine may be a promising adjunctive treatment for schizophrenia, potentially improving various domains including quality of life, cognitive function, negative symptoms, and depressive symptoms. However, larger and more robust studies are needed to confirm these findings.

**Disclosure of Interest:**

None Declared

